# UNIQUE STRATEGIES AT CEDARS-SINAI TO SCREEN FOR AND ADDRESS SOCIAL DETERMINANTS OF HEALTH IN OLDER ADULTS

**DOI:** 10.1093/geroni/igae098.2881

**Published:** 2024-12-31

**Authors:** Sonja Rosen, Emma Cohen, Katie Hren, Christina Harris, Krystal Green

**Affiliations:** Cedars-Sinai Medical Center, Los Angeles, California, United States; Cedars-Sinai Medical Center, Los Angeles, California, United States; Cedars-Sinai Medical Center, Los Angeles, California, United States; Cedars-Sinai Medical Center, Los Angeles, California, United States; Cedars-Sinai Medical Center, Los Angeles, California, United States

## Abstract

To effectively address health disparities and bridge systemic gaps between health and social care, healthcare institutions must cohesively address shortcomings in identifying, supporting, and monitoring social determinants of health (SDoH) to better serve communities. In 2021, Cedars-Sinai, a Los Angeles urban Academic Medical Center, launched a robust strategy to address SDoH needs across health system settings. Cedars-Sinai’s SDoH strategy includes a customized 12-domain screener to identify patients unmet social care needs and additional workstreams to support patient and community needs, including an electronic resource referral platform, an inaugural Community Health Worker (CHW) program, and a network of community-based organizations (CBOs) with accountable referral pathways. As one of the largest Medicare providers in the region, and serving more older adults over the age of 80 than any other academic medical center, this SDoH strategy included tailored interventions for older adults such as embedding a CHW within the geriatrics outpatient practice and cultivating community partnerships with CBOs experienced in serving older adults. In the first 2.5 years of Cedars-Sinai’s SDoH strategy, 97,757 older adult patient screenings identified 30,245 unmet HRSN needs; 411 of these older adult patients received CHW support, 61.24% of whom were successfully connected to resources. These systemwide efforts highlighted that older adults experience more nuanced SDoH inequalities fueled by aging-specific structural barriers and succeed with greater dosages of social care interventions. Findings from Cedars-Sinai’s approach to SDoH has demonstrated that healthcare institutions are most effective in addressing SDoH through comprehensive strategies that create population-specific solutions in tandem with screening.

